# Granzyme B: A Double-Edged Sword in the Response to Influenza Infection in Vaccinated Older Adults

**DOI:** 10.3389/fragi.2021.753767

**Published:** 2021-11-11

**Authors:** Chris P. Verschoor, Graham Pawelec, Laura Haynes, Mark Loeb, Melissa K. Andrew, George A. Kuchel, Janet E. McElhaney

**Affiliations:** ^1^ Health Sciences North Research Institute, Sudbury, ON, Canada; ^2^ Northern Ontario School of Medicine University, Sudbury, ON, Canada; ^3^ Department of Immunology, University of Tübingen, Tübingen, Germany; ^4^ UConn Center on Aging, University of Connecticut School of Medicine, Farmington, CT, United States; ^5^ Department of Pathology and Molecular Medicine, McMaster University, Hamilton, ON, Canada; ^6^ Department of Medicine, Dalhousie University, Halifax, NS, Canada

**Keywords:** influenza, granzyme B, older adults, influenza vaccination, laboratory-confirmed influenza illness, T cell responses

## Abstract

**Background:** Influenza-specific cytolytic T lymphocytes (CTL) have a critical role in clearing the virus from the lungs, but are poorly stimulated by current inactivated influenza vaccines. Our previous work suggests that granzyme B (GrB) activity predicts protection against laboratory-confirmed influenza infection (LCII) in older adults. However, basal GrB (bGrB) activity increases with age and the frequency of GrB^+^ CTL that do not co-express perforin increases following influenza infection, thereby acting as a potential contributor to immune pathology.

**Objectives:** Using data from a 4-years randomized trial of standard-versus high-dose influenza vaccination, we sought to determine whether measurements of GrB activity alone indicate a protective vs pathologic response to influenza infection. We compared LCII to No-LCII subsets according to: pre-vaccination bGrB activity; and induced GrB activity in *ex vivo* influenza-challenged peripheral blood mononuclear cells (PBMC) at four and 20weeks post-vaccination.

**Results:** Over four influenza seasons (2014–2018), 27 of 608 adult participants aged 65 years and older developed influenza A/H3N2-LCII (n = 18) or B-LCII (n = 9). Pre-vaccination, there was a significant correlation between bGrB and *ex vivo* GrB activity in each of the H3N2-LCII, B-LCII, and No-LCII subsets. Although pre-vaccination *ex vivo* GrB activity was significantly higher in B-LCII vs No-LCII with a trend for H3N2-LCII vs No-LCII, there was no difference in the response to vaccination. In contrast, there was a trend toward increased pre-vaccination bGrB activity and LCII: Odds Ratio (OR) (95% confidence intervals) OR = 1.46 (0.94, 2.33). By 20-weeks post-vaccination, there were significant fold-increases in *ex vivo* GrB activity specific for the infecting subtype in H3N2-LCII: OR = 1.63 (1.35, 2.00) and B-LCII: OR = 1.73 (1.34, 2.23).

**Conclusions:** Our results suggest that the poor GrB responses to influenza vaccination that led to development of LCII can be attributed to inactivated formulations rather than the aging immune system since LCII cases generated robust *ex vivo* GrB responses following natural infection. Further, we identified bGrB as a biomarker of those who remain at risk for LCII following vaccination. Future studies will focus on understanding the mechanisms responsible for the shift in GrB-mediated protection vs potential immune pathology caused by GrB release.

## Introduction

Despite widespread vaccination programs, the burden of influenza illness continues to be high in older adults, among whom increasing frailty is associated with declines in vaccine effectiveness for the prevention of hospitalization (Andrew et al., 2017). Influenza hospitalization rates are highest in seasons when influenza A/H3N2 strains are circulating (2017–2018) and during the 2017–2018 influenza season when A/H3N2 was the predominant circulating strain, there was a 1.6-fold increase over an average year with 953,000 hospitalizations, and 79,400 deaths associated with influenza in the United States (Rolfes et al., 2019). Although it is well known that the generation of strain-specific antibodies are important for preventing viral entry, their predictive ability in the response to influenza vaccination is quite limited in terms of risk of infection, and cell-mediated immune responses are needed for protection against serious complications once infection occurs.

Protection from severe influenza disease relies on cytolytic CD8^+^ T cells to clear influenza virus from the lungs (McMichael et al., 1983; Grant et al., 2016; Jansen et al., 2019), thus also limiting the inflammatory response to infection (van den Berg et al., 2021) that leads to cardiovascular complications including strokes (Smeeth et al., 2004; Toschke et al., 2009), heart failure (Panhwar et al., 2019; Vardeny and Solomon, 2019), and ischemic heart disease (Smeeth et al., 2004; Barnes et al., 2015; Kwong et al., 2018; Cardoso et al., 2020). Critical to the function of cytolytic T cells is granzyme B (GrB), a serine protease secreted together with the pore-forming molecule perforin (Perf), which facilitates GrB entry into target cells, leading to their apoptosis (Voskoboinik et al., 2015). We have used an *ex vivo* influenza challenge model in human peripheral blood mononuclear cells (PBMC) to simulate the cell-mediated immune response to natural infection and have shown that the level of GrB activity in lysates of A/H3N2 challenged PBMC correlates with protection against laboratory-confirmed influenza A/H3N2 infection (LCII) in older adults (McElhaney et al., 2006; McElhaney et al., 2009; Shahid et al., 2010). Further, the decline in vaccine effectiveness with age corresponds to a decreased frequency of GrB^+^Perf^+^ CD8^+^ T cells and related cytolytic activity following influenza vaccination in older compared to young adults (Zhou and McElhaney, 2011; Zhou et al., 2016). We then discovered that overall GrB activity in circulating T cells of older adults was substantial and that the level of this “basal” GrB (bGrB) activity: 1) increases with age and in frail compared to non-frail older adults (Verschoor et al., 2021b); 2) is inversely proportional to the frequency of memory CD8^+^ T cells; and 3) is proportional to the frequency of circulating late- or terminally-differentiated CD8^+^ T cells some of which may be truly senescent (Haq et al., 2017); our unpublished data has shown a similar correlation between bGrB activity and the frequency of circulating CD8^+^ T cells that express GrB in the absence of Perf. Further, upon *ex vivo* infection with influenza A/H3N2 virus, ∼50% of the total CD8^+^ T cell population express GrB in the absence of Perf (Kumar et al., 2017) and can play an important role in immune pathology resulting in the loss of tissue function and integrity (Choy et al., 2004; Tschopp et al., 2006; Granville, 2010; Hendel et al., 2010; Russo et al., 2018; Turner et al., 2019; Matsubara et al., 2020). In contrast, only 2–3% CD8^+^ T cells co-express GrB and Perf (Kumar et al., 2017) and have targeted cytolytic activity specifically against influenza-infected PBMC (Zhou and McElhaney, 2011; Zhou et al., 2016).

In this 4-year randomized trial, we tested the ability of our standard operating procedures for *ex vivo* stimulation of PBMC with standardized preparations of live influenza virus and a validated assay of GrB activity, to predict risk for LCII in vaccinated older adults. We have previously shown that bGrB activity is increased in older compared to young adults and that the fold-increase in GrB activity above this baseline (bGrB) activity, induced with *ex vivo* challenge is diminished in older compared to young adults (Verschoor et al., 2021b). In this study, we explored bGrB activity pre-vaccination and GrB activity in *ex vivo* virus-challenged PBMC at 4-weeks post-vaccination as predictors of a subsequent LCII, and how *ex vivo* stimulated GrB activity changed following LCII at 20-weeks post-vaccination. We found that bGrB activity was a predictor of risk for LCII while the *ex vivo* induced GrB activity response to influenza vaccination was not. Our results underscore the importance of determining the source of GrB activity from T cells that co-express perforin with GrB (i.e., protective cytolytic activity) vs those T cells that do not express perforin and release GrB into the extracellular space in response to influenza infection potentially contributing to immune pathology.

## Materials and Methods

### Study Design

**FIGURE 1:**
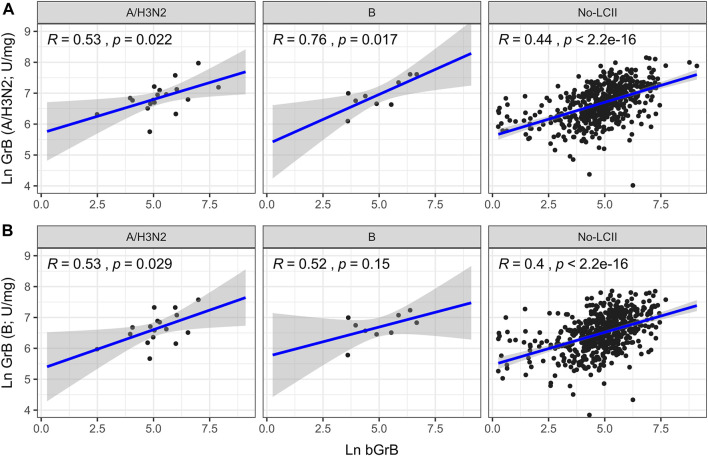
Correlation of natural-log (Ln) basal granzyme B activity (bGrB) with total GrB in response to **(A)** H3N2 and **(B)** B virus prior to vaccination. Trend line and confidence interval (shaded area) and individual data points shown for participants who developed A/H3N2 (n = 18) and B (n = 9) LCII and those who didn’t (ie. Null; n = 582), along with Pearson’s correlation (R) and test significance (p).

This study was conducted to compare bGrB activity at the study baseline and GrB activity in *ex vivo* influenza challenged PBMC at 4-weeks post-vaccination in older adults who went on to develop LCII as part of a randomized, double-blind trial of a Fluzone^®^ HD-SVV vs SD-SVV in community-dwelling older adults (See details at ClinicalTrials.gov: NCT02297542). In this trial, participants enrolled in previous years were eligible for enrollment and re-randomization in subsequent years as in the parent trial (DiazGranados et al., 2014) and GrB responses to *ex vivo* influenza A/H3N2 or B were measured pre-vaccination and 4, 10 and 20-weeks post-vaccination over four influenza seasons (October 2014—April 2015, October 2015—April 2016, October 2016—April 2017, and October 2017—April 2018) as previously described (Loeb et al., 2020). To this end, a total of 582 older adults was enrolled over the four seasons as previously reported (see Figure 1 in (Loeb et al., 2020)). Those who developed LCII were not excluded from the trial in subsequent years. The study protocol was approved by the Institutional Review Board of the University of Connecticut Health Centre and the Health Sciences North Research Ethics Board (Sudbury, ON, Canada) and all study participants provided written informed consent prior to participation in the study.

### Participant Recruitment and Characterization

Older adults (aged 65 years and older) were recruited through the University of Connecticut Center on Aging Recruitment Core (UCARC) and through the Health Sciences North Research Institute (HSNRI) as previously described (Loeb et al., 2020). Inclusion criteria: adults ≥ 65 years old who were vaccinated in the previous influenza season. Exclusion criteria: 1) known immunosuppressive disorders or medications; 2) a previous severe reaction to the vaccine components or refusal of vaccination; and 3) prior influenza vaccination in the community for the approaching influenza season. In the case of any acute respiratory illness at the time of enrollment, vaccinations were scheduled at least 2 weeks later.

Following informed consent, study participants were characterized according to demographic data [age, sex, ethnicity and body-mass index (BMI)], chronic medical conditions, including known risk factors for influenza illness (pulmonary, cardiac, metabolic, renal, or neoplastic disorders), health attitudes, symptoms, and functional impairments. A Frailty Index (FI) was calculated based on 40 items validated in terms of outcomes of influenza (Theou et al., 2020). The FI was included in regression analyses as a continuous variable to capture the full spectrum of frailty in the sample, and was categorized using validated cutoffs [ie. robust (FI < 0.10), pre-frail (FI = 0.10–0.21) and frail (FI > 0.21)] to describe the sample in terms of clinically distinct groups.

### Influenza Vaccination

Older adult participants were randomized 1:1 to receive either the trivalent, split-virus Fluzone SD vaccine (15 μg of HA per strain) (n = 296) or Fluzone HD vaccine (60 μg of HA per strain) (n = 286). The following strains were contained in the vaccine each year: 2014/2015: A/California/7/2009 (H1N1)-like virus, A/Texas/50/2012 (H3N2)-like virus and B/Massachusetts/2/2012-like virus; 2015/2016: A/California/7/2009 (H1N1)pdm09-like virus, A/Switzerland/9715293/2013 (H3N2)-like virus, and B/Phuket/3073/2013-like virus; 2016–2017: A/California/7/2009 (H1N1)pdm09-like virus, A/Hong Kong/4801/2014 (H3N2)-like virus, B/Brisbane/60/2008-like virus; 2017–2018: A/Michigan/45/2015 (H1N1)pdm09-like virus; A/Hong Kong/4801/2014 (H3N2)-like virus; and B/Brisbane/60/2008-like virus. Clinical and laboratory research staff remained blinded to the vaccine group until the study database for each year was unlocked.

### Influenza Surveillance

Influenza surveillance was initiated in November of each year using local surveillance data. Intensive monitoring for acute respiratory infection (ARI), with weekly phone calls to study participants, began when influenza was confirmed to be circulating in the local community and continued through April of the following year. Intensive ARI monitoring included weekly contact with study participants to assess for any flu-like symptoms or ARI, and included nasopharyngeal swabs (within 5 days of onset of symptoms) for RT-PCR detection of influenza. This intensive monitoring allows participants to report symptoms early, so that a potential influenza illness is identified, and referral made to their primary care physician for follow-up. ARI included upper (coryza or sore throat) or lower (cough or shortness of breath) respiratory tract symptoms, or headache, malaise, myalgia or fever (>99°F or 37.3°C orally). LCII was defined as a positive polymerase chain reaction (PCR) test for influenza pH1N1, A/H3N2 or B or a 4-fold or greater rise in antibody titer following infection (see below). No-LCII participants were defined as those who did not develop LCII in that influenza season.

### Blood Collection and Isolation of PBMCs

Blood samples (35 cc heparinized whole blood and 5 cc of serum) were collected at the pre-vaccination and 4, 10 and 20-weeks post-vaccination visits. PBMCs were isolated from heparinized blood samples on the day of collection using Ficoll-Paque Plus (GE Healthcare) gradient purification, frozen in 90% human AB sera and 10% DMSO, and transferred to liquid nitrogen for storage. Serum samples were frozen at −80°C.

### Serum Antibody Titers

HAI assays were performed using a single stock source (Centers for Disease Control) of HA for each of the vaccine strains pre-vaccination and 4, 10 and 20 weeks post-vaccination. HAI were performed in 2-fold dilutions of serum from 1/10 to 1/1024 and titers <1/10 are calculated as 1/10. Titres have been previously published (Verschoor et al., 2021a), but can also be found in the supplementary materials (Supplemental Table S1). LCII was defined as a 4-fold rise in serum HAI titers from 4-weeks to 20-weeks post-vaccination.

### *Ex vivo* Influenza Challenge

PBMCs were thawed, rested overnight, and then stimulated with live influenza A/H3N2 or B virus (sucrose-gradient purified A/Victoria/3/75 (H3N2) or B/Lee/40, Charles River) at a multiplicity of infection of two in AIM V media incubated at 37°C/5%CO_2_ for 20 h according to our Standard Operating Procedure (SOP) (McElhaney and Gentleman, 2015). These influenza virus strains have been chosen for consistency across multi-year studies and contain hemagglutinin and internal protein (matrix one and nucleoprotein) peptide sequences that are shared and thus cross-reactive across all A/H3N2 or B strains.

### Granzyme B Activity Assay

This assay capitalizes on the enzyme’s unique substrate specificity for cleavage at the aspartate residue of IEPDpna and the release of paranitroanilide (pna) (Gijzen et al., 2010), and correlates with cytotoxicity (by ^51^Cr-release assays) in influenza-stimulated PBMC (McElhaney et al., 1996; Ewen et al., 2003). Assays of GrB activity in A/Victoria/3/75 (H3N2)-stimulated PBMC have been validated across multiple laboratories according to the International Council for Harmonisation of Technical Requirements for Pharmaceuticals for Human Use (ICH) (Gijzen et al., 2010) Importantly, minimal assay-to-assay variability makes possible the comparison of results from one study year to the next (McElhaney et al., 2009), and across laboratories (Shahid et al., 2010) and was performed under our SOP (McElhaney and Gentleman, 2015). Basal GrB (bGrB) activity was measured in lysates of CD3^+^ T cells isolated from peripheral blood obtained at baseline. GrB activity was measured in lysates of A/Victoria/3/75 (H3N2) or B/Lee/40-stimulated PBMC obtained at all time points. Induced GrB (iGrB) was calculated as the log (*ex vivo* GrB activity)—log (bGrB activity).

### Statistical Analysis

For participant demographics, continuous data were summarized as the mean and standard deviation, and categorical data as the count and frequency. For GrB activity measures, they were summarized as the geometric mean and 95% confidence interval. For comparisons between LCII and No-LCII subsets according to bGrB activity pre-vaccination, and *ex vivo* GrB activity against the influenza A/Victoria (H3N2) and B/Lee challenge strains prior to (4 weeks post-vaccination) and following LCII (20-weeks post-vaccination), overlapping confidence intervals indicated no statistically significant differences. The correlation between bGrB and *ex vivo* GrB activity in each of the LCII and No-LCII subsets was evaluated according to the Pearson’s correlation co-efficient (R value) and test of significance (*p* value). To compare the change in GrB activity from four to 20-weeks between LCII and No-LCII groups we performed mixed model linear regression, where the natural-log week-20 activity was regressed on the fixed effects of natural-log week-4 activity, frailty index and LCII status, and random intercepts for participant and year. These variables were chosen based on minimization of the Akaike’s Information Criterion (ie. AIC). To determine if GrB activity at baseline or 4-weeks post-vaccination, the fold-change in GrB from baseline to 4-weeks or bGrB activity was associated with the likelihood of LCII, mixed model logistic regression was performed where LCII status was regressed on the standardized (mean = 0, standard deviation = 1) natural-log bGrB, GrB or GrB fold-change measure, adjusting for the random intercept of year. Findings are presented as the odds ratio and 95% confidence interval. All demographic variables were considered as covariates in the model, but only year was found to improve the AIC, and standardization was performed in order to facilitate comparability across the different measures. For models including GrB, parallel analyses were performed: one in which activity estimates for participants that did not develop LCII were based on *ex vivo* stimulation with A/Victoria (H3N2) virus and the other where they were based on *ex vivo* stimulation with B/Lee virus. For both analyses, activity estimates for participants that developed A/H3N2 LCII were based on *ex vivo* stimulation with A/Victoria (H3N2) virus, whereas estimates for participants that developed B-LCII were based on *ex vivo* stimulation with B/Lee virus.

## Results

### Influenza Surveillance and Participant Characteristics

Over four influenza seasons, 30 LCII cases (HD vaccine n = 10 cases, SD vaccine n = 20 cases) were detected (as previously reported (Verschoor et al., 2021a)) and corresponded to hospitalization rates for the circulating strains as reported by the Centers for Disease Control Weekly United States Influenza Surveillance Report (FluView) for that influenza season. The majority of these cases occurred in year 4 (2017–2018, n = 9 H3N2-LCII and n = 6 B-LCII) when a vaccine strain mismatch to the circulating A/H3N2 occurred in the process of egg adaptation of the virus for vaccine production and led to an earlier influenza season (i.e., prior to the 10-weeks post-vaccination time point) and record-breaking hospitalization rates in the 65+ population. The next largest number occurred in year 1 (n = 7 H3N2-LCII) when antigenic drift in the circulating A/H3N2 strain resulted in a vaccine strain mismatch and was associated with increased hospitalization rates in the 65+ population. Participants that developed A/H1N1-LCII (n = 3) were removed from further analysis due to the small number of cases.

LCII and non-LCII participants (See Table 1) were nearly identical with respect to age (mean ± standard deviation: 76 ± 7.4), BMI (28 ± 4.8), level of frailty (50% robust, 41% pre-frail, and 9% frail) and CMV serostatus (47% seronegative). Although not statistically significant, LCII vs non-LCII participants were more likely to be male vs female (frequency: A/H3N2 = 50:50; B = 56:44; Null = 32:68%), have received the SD vaccine (A/H3N2 = 67:33; B = 67:33; Null = 51:49%), and for B cases, to be enrolled at the HSNRI site (B = 100:0; Null = 58:49%).

**TABLE 1:** Demographics.

		LCII at 8–20 weeks		
	Total	A/H3N2	B	Null
	(N = 609)	(N = 18)	(N = 9)	(N = 582)
**Age**	76 (7.37)	76.2 (6.2)	78.1 (7.46)	76 (7.41)
**Sex**	—	—	—	—
Female	408 (67%)	9 (50%)	4 (44%)	395 (68%)
Male	201 (33%)	9 (50%)	5 (56%)	187 (32%)
**Body-mass index**	27.9 (4.84)	27.8 (5.19)	29.8 (4.12)	27.9 (4.84)
Missing	3 (0.5%)	0 (0%)	0 (0%)	3 (0.5%)
**CMV serostatus**	—	—	—	—
Negative	285 (47%)	10 (56%)	4 (44%)	271 (47%)
Positive	324 (53%)	8 (44%)	5 (56%)	311 (53%)
**Frailty category**	—	—	—	—
Robust	304 (50%)	7 (39%)	4 (44%)	293 (50%)
Pre-frail	249 (41%)	9 (50%)	5 (56%)	235 (40%)
Frail	54 (9%)	2 (11%)	0 (0%)	52 (9%)
Missing	2 (0.3%)	0 (0%)	0 (0%)	2 (0.3%)
**Dose**	—	—	—	—
HD	295 (48%)	6 (33%)	3 (33%)	286 (49%)
SD	314 (52%)	12 (67%)	6 (67%)	296 (51%)
**Site**	—	—	—	—
HSNRI	354 (58%)	10 (56%)	9 (100%)	335 (58%)
UCHC	255 (42%)	8 (44%)	0 (0%)	247 (42%)

### Correlation Between Basal Granzyme B and *ex vivo* Granzyme B Activity at Baseline

Because we know that the percentage of GrB^+^Perf^−^ CD8^+^ cells greatly outweighs the GrB^+^Perf^+^ cells, most of the GrB measured must be originating from these Perf^−^ cells. Thus, we compared the correlation between bGrB and *ex vivo* GrB activity across the different LCII and no-LCII subsets. We found a higher correlation between bGrB and *ex vivo* GrB activity (A/Victoria (H3N2) or B/Lee-challenged PBMC) in the LCII (R range: 0.52–0.76) compared to No-LCII (R range: 0.4–0.44) subsets (Figure 1).

### Granzyme B Activity as a Predictor of and Response to H3N2- and B-Laboratory-Confirmed Influenza Illness

**FIGURE 2:**
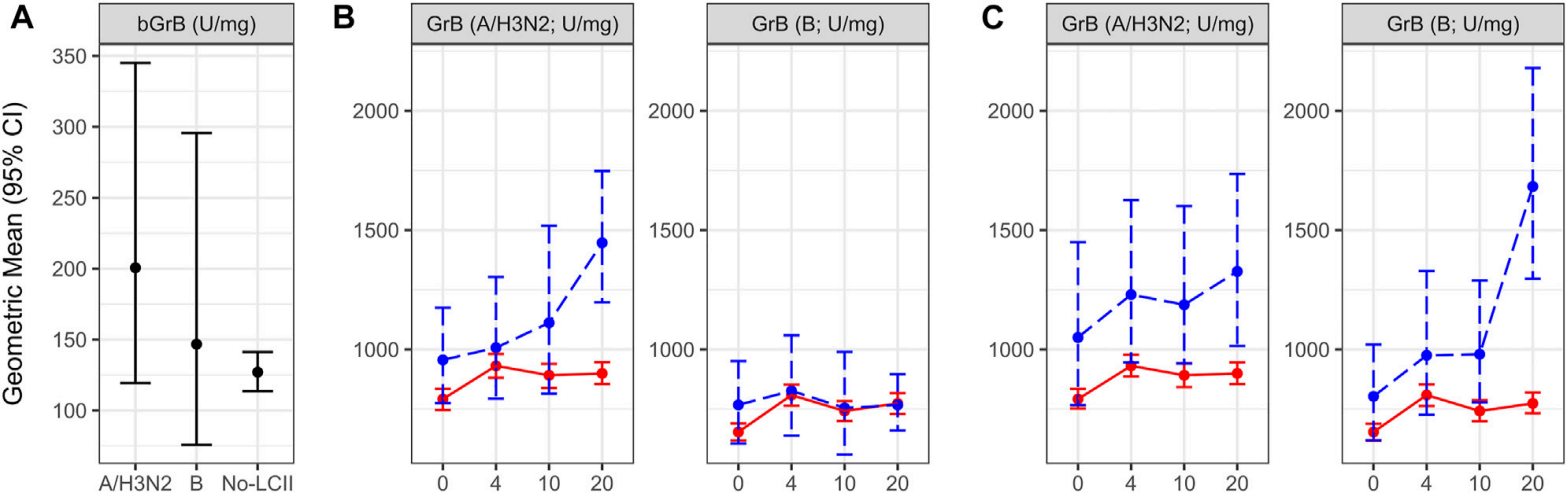
Change over the study period for granzyme B activity (GrB) in PBMCs, and for basal GrB activity (bGrB) stratified by laboratory-confirmed influenza illness (LCII) status at 4–20 weeks post-vaccination compared to the Null (No-LCII) subset. **(A):** The geometric mean and 95% CI for bGrB is presented for each LCII status category GrB activity in response to *ex vivo* stimulation with **(B):** A/Victoria **(left)** or B/Lee **(right)** virus in H3N2 LCII vs Null subsets and **(C):** A/Victoria **(left)** or B/Lee **(right)** virus in B-LCII vs Null subsets. GrB activity levels at pre-vaccination (0 weeks) and 4, 10 and 20-weeks post-vaccination are presented as the geometric mean and 95% confidence interval. For both **B** and **C**, participants without documented LCII are indicated by red, solid lines (n = 582); blue, dashed lines indicate participants with A/H3N2 (n = 18; panel **B**), and B LCII (n = 9; panel **C**).

Prior to influenza vaccination, geometric mean bGrB activity tended to be higher in H3N2-LCII: 201 [95% confidence interval (CI) 120, 336] compared to B-LCII: 147 (73, 305) and No-LCII 127 (114, 141) participants (Figure 2A). In contrast, by 4-weeks post-vaccination there was no difference in geometric mean GrB activity measured in A/Victoria (H3N2)-stimulated PBMC in H3N2-LCII: 1007 (779, 1309) vs No-LCII: 931 (885, 982) subsets (Figure 2B, left panel; Supplemental Table S2) and similar results were observed for B/Lee-stimulated GrB activity in H3N2-LCII: 826 (650, 1051) vs No-LCII: 809 (766, 853) (Figure 2B, right panel; Supplemental Table S2). Following natural infection, H3N2-LCII cases compared to No-LCII participants showed a significant increase in A/Victoria (H3N2)-stimulated GrB activity at 20-weeks post-vaccination; H3N2-LCII: 1447 (1198, 1745) vs No-LCII: 899 (854, 948) (Figure 2B, left panel; Supplemental Table S2; *p* < 0.001); but not B/Lee-stimulated GrB activity in H3N2-LCII: 767 (651, 904) vs No-LCII: 774 (734, 815) subsets (Figure 2B, right panel; Supplemental Table S2), consistent with an influenza A type-specific GrB response to natural infection.

In contrast, the B-LCII compared to the No-LCII subset tended to show higher GrB activity levels at 4-weeks post-vaccination with both A/Victoria (H3N2) stimulation in B-LCII: 1230 (940, 1574) vs No-LCII: 931 (885, 982) PBMC (Figure 2C, left panel, Supplemental Table S2) and B/Lee stimulation in B-LCII: 975 (722, 1357) vs No-LCII: 809 (766, 853) PBMC (Figure 2C, right panel, Supplemental Table S2). Similar to the influenza type-specific response following natural infection with A/H3N2, B-LCII cases showed a significant increase in B/Lee-stimulated GrB activity compared to the No-LCII subset at 20-weeks post-vaccination; B-LCII: 1683 (1272, 2194) vs No-LCII: 774 (734, 815) (Figure 2C, right panel; Supplemental Table S2; *p* < 0.001). Compared to the No-LCII subset, B-LCII cases showed persistently elevated GrB activity at all time points in A/Victoria (H3N2)-stimulated PBMC with no significant increase following influenza B infection; B-LCII: 1327 (976, 1724) vs No-LCII: 899 (854, 948) GrB activity was observed at 20-weeks post-vaccination (Figure 2C, left panel, Supplemental Table S2). This latter observation in B-LCII PBMC may be related to prior seasonal exposure to influenza A/H3N2 infection in some of the B-LCII cases (McElhaney et al., 2009).

### Granzyme B Response to Vaccination as a Predictor of Risk for Laboratory-Confirmed Influenza Illness

**FIGURE 3:**
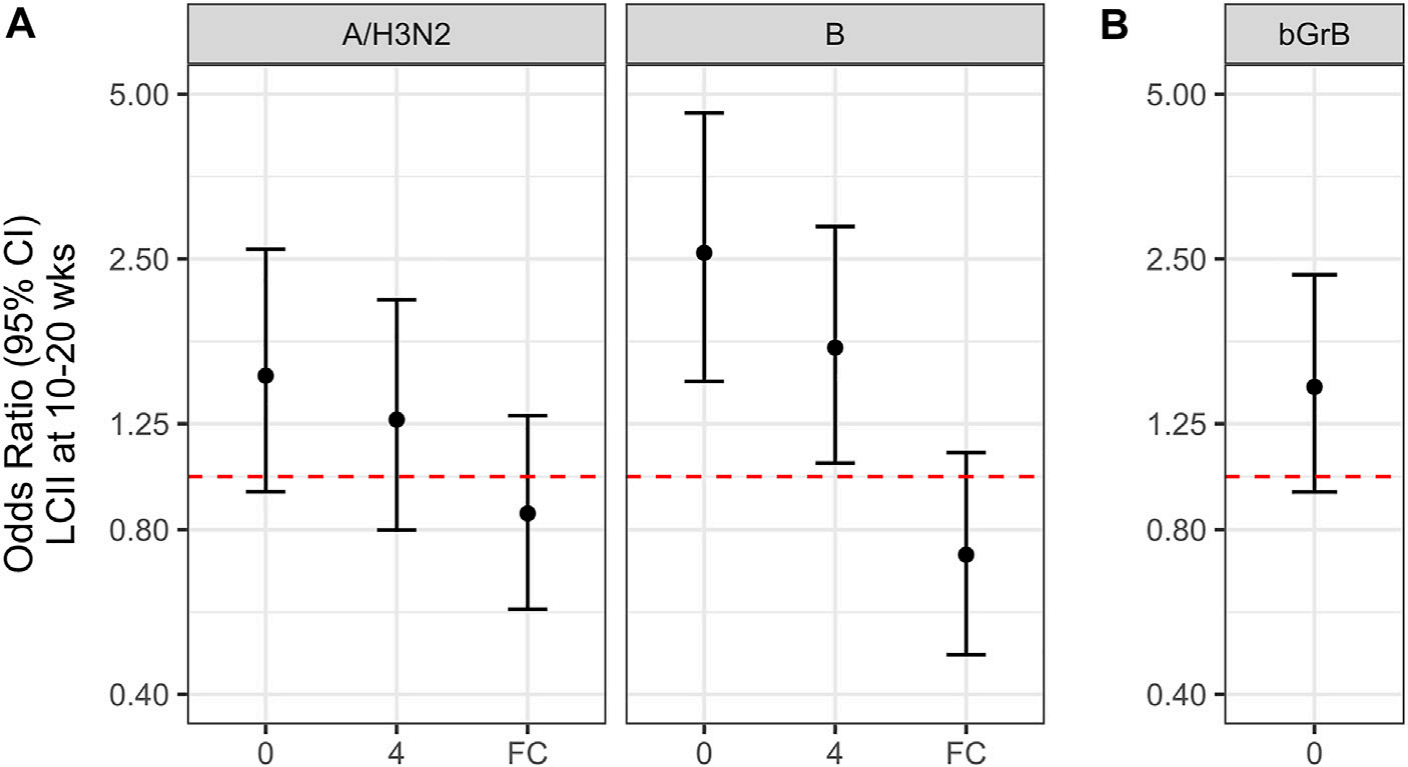
The likelihood of laboratory confirmed influenza illness (LCII) at 10–20 weeks post-vaccination, **(A),** relative to *ex vivo* granzyme B (GrB) activity, and **(B),** basal GrB (bGrB) activity in circulating T cells. The odds ratio (OR) and 95% confidence interval for being LCII positive per 1-standard deviation in the natural-log of each measure at pre-vaccination (0-weeks) or 4-weeks post-vaccination or the natural-log fold-change (FC) pre-to 4-weeks. For both **A** and **B**, activity estimates for participants that developed A/H3N2 LCII **A (left panel)** were based on *ex vivo* stimulation with A/Victoria (H3N2) virus, whereas estimates for participants that developed B-LCII **A (right panel)** were based on *ex vivo* stimulation with B/Lee virus; the corresponding estimates for No-LCII participants from A/Victoria (H3N2) stimulation **A (left panel)** and from B/Lee stimulation **A (right panel)** were used to calculate the OR. Overlap between the confidence interval and the red, dashed line (ie. OR = 1) indicates that the odds ratio is not statistically significant.

To evaluate GrB activity and the response to vaccination as a predictor of risk for influenza illness, we determined the likelihood of LCII relative to the standardized pre- and 4-weeks post-vaccination activity levels and the fold-change in *ex vivo* GrB activity with vaccination. For this, we performed mixed model logistic regression where LCII status was regressed on the standardized natural-log bGrB, GrB or GrB fold-change measure, adjusting for the random effect of year. The odds ratio for LCII was significantly increased in the B-LCII compared to the No-LCII subset in *ex vivo* B/Lee-challenged PBMC at pre- and 4-weeks post-vaccination, where a 1-standard deviation increase in GrB at pre- and 4-weeks post-vaccination increased the odds of LCII by 2.57 (95% CI, 1.49–4.62) and 1.72 (95% CI, 1.06–2.87), respectively (Figure 3A, right panel). There was a similar trend in H3N2-LCII vs No-LCII PBMC challenged with A/Victoria (H3N2) (Figure 3A, right panel), but the odds ratio in terms of the response to vaccination in either H3N2- or B-LCII cases was not significant. Lastly, there was an increased bGrB associated with a greater odds of LCII by 1.46 (95% CI, 0.94–2.34) but this was also not statistically significant (Figure 3B).

### Inducible Granzyme B With *ex vivo* Stimulation Following Vaccination and Infection

**FIGURE 4:**
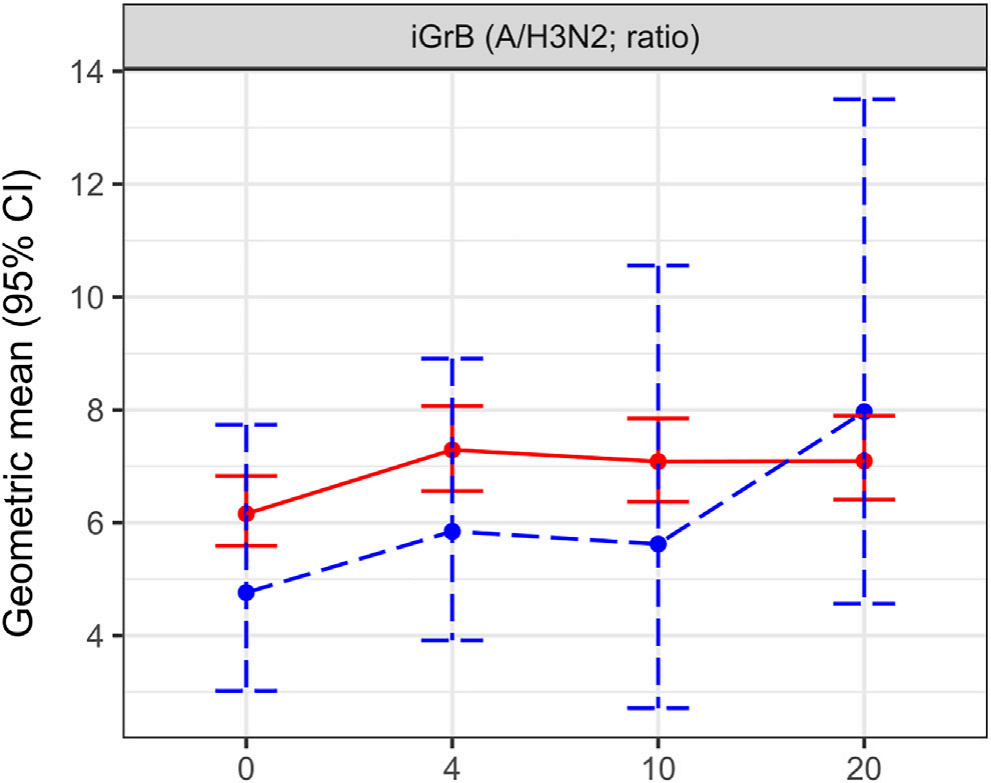
Change over the study period for inducible granzyme B (iGrB) levels in A/Victoria (H3N2) challenged PBMCs stratified by H3N2 laboratory-confirmed influenza illness (H3N2-LCII) status at 10–2 weeks post-vaccination compared to the Null (No-LCII) subset. iGrB levels at pre-vaccination (0 weeks) and 4, 10 and 20-weeks post-vaccination are presented as the geometric mean and 95% confidence interval. Participants without documented LCII are indicated by red, solid lines (n = 582); blue, dashed lines indicate participants with H3N2-LCII (n = 19).

We have also calculated the amount of GrB activity induced with ex vivo stimulation (iGrB) as log (iGrB) = log (ex vivo GrB)—log (bGrB) according to our published results from this clinical trial, which showed an age-related decline in iGrB levels in older compared to young adults included as a control group (Verschoor et al., 2021b). Although there were too few LCII cases to determine differences in iGrB levels in H3N2-LCII and No-LCII at 4-weeks post-vaccination, we did observe a poor iGrB response to vaccination in the H3N2-LCII subset but a significant increase in iGrB levels following H3N2 infection (Figure 4), consistent with re-stimulation of CTL memory following infection.

## Discussion

In a longitudinal study of the response to influenza vaccination in older adults, we sought to determine whether measurements of GrB activity in resting T cells and under conditions of *ex vivo* challenge with live influenza virus were informative with regards to protection against LCII. Prior to vaccination, we found a significant correlation between basal GrB activity in resting T cells and *ex vivo* stimulated GrB activity in each of the H3N2-LCII, B-LCII and corresponding No-LCII subsets. This bGrB activity tended to be higher in H3N2-LCII cases compared to B-LCII cases and the corresponding No-LCII subsets. In contrast, GrB activity in *ex vivo* influenza A/Victoria (H3N2) or B/Lee-challenged PBMC was consistently higher at all time points in the B-LCII subset compared to the H3N2-LCII and corresponding No-LCII subsets. A regression analysis confirmed increased odds of B-LCII based on increased levels of *ex vivo* GrB activity in B/Lee-stimulated PBMC at pre- and 4-weeks post-vaccination but the change in *ex vivo* GrB activity in response to vaccination did not predict risk for H3N2- or B-LCII. When adjusted for the effect of bGrB activity in the calculation of iGrB, H3N2-LCII cases showed a poor iGrB response to influenza vaccination but a robust iGrB response to A/Victoria (H3N2) but not B/Lee following influenza infection.

Historically, influenza vaccines have been designed to stimulate protective antibody responses to the critical epitopes surrounding the receptor-binding domain of the globular head of hemagglutinin (HA) that permits infection of the host cell. However, when vaccines fail to provide sterilizing antibody-mediated protection against infection, cytotoxic T lymphocytes (CTL) are needed to clear the virus from the lungs once infection occurs. CTL mediate protection against disease in older adults (Rimmelzwaan and McElhaney, 2008) and against disease in young adults with low antibody titers (Wilkinson et al., 2012; Sridhar et al., 2013). However, inactivated influenza vaccines provide only a weak stimulus to CD8^+^ T cell memory and translate to the poor induced GrB response to influenza vaccination and breakthrough LCII in vaccinated older adults that we have observed in multiple studies (McElhaney et al., 2001; McElhaney et al., 2006; McElhaney et al., 2009; Shahid et al., 2010).

CTL recognizing the conserved epitopes of influenza internal proteins such as matrix (M1) and nucleoprotein (NP) contribute to heterosubtypic immunity which limits the spread of the virus in the respiratory tract and prevents lethal viral pneumonia—this has been shown to be critical for broad protection against all influenza A strains (Jang and Seong, 2019). To simulate this response to natural infection, we have developed an *ex vivo* model using a sucrose-gradient purified, standardized preparation of A/Victoria/3/75 (H3N2) or B/Lee/40, which contain the internal M1 and NP proteins that are shared across all influenza A or influenza B strains. Upon infection, host cells present peptides derived from viral proteins in the process of viral replication, and loaded onto Major Histocompatibility Complex I (MHCI) molecules. These peptide/MHCI epitopes are recognized by and provide a strong stimulus to activate influenza-specific memory CD8^+^ T cells. Upon binding to their specific target cells, Perf and GrB are released by CD8^+^ T cells into the immunologic synapse wherein Perf facilitates the directed entry of GrB and mediates protection by killing influenza-infected host cells. Employing this *ex vivo* model of influenza challenge using standardized strains of influenza, we have found that *ex vivo* GrB activity correlates with protection against A/H3N2 illness in multiple studies of largely healthy older adults (McElhaney et al., 2006; McElhaney et al., 2009; Shahid et al., 2010). However, we now recognize that there are two populations of CD8^+^ T cells that contribute to the GrB activity measured in *ex vivo* influenza challenged PBMC, those that co-express GrB and Perf and those that produce GrB in the absence of Perf (Kumar et al., 2017). Since increased bGrB activity predicted LCII in this study, we postulate that CD8^+^ T cells that are the source of bGrB, express GrB in the absence of Perf when activated in response to influenza infection.

Following our discovery of GrB activity in circulating T cells, we found that this bGrB activity increased with age, frailty (Verschoor et al., 2021b) and cytomegalovirus (CMV) seropositivity, and correlated with the frequency of late/terminally-differentiated, potentially senescent, CD8^+^ T cells (Haq et al., 2017; McElhaney et al., 2020). A recent study showed that CMV seropositivity in older adults was associated with 1) an increased frequency of CD8^+^ T cells expressing senescence-associated cell surface markers; 2) lower frequencies of influenza-specific memory T cells; and 3) an enhanced inflammatory response to influenza infection, but no difference in the memory T cell response to influenza infection when compared to seronegative individuals (van den Berg et al., 2021). In our studies, we noted a high proportion of GrB^+^Perf^−^CD8^+^ T cells in *ex vivo* A/Victoria (H3N2)-infected PBMC that was not observed in CD4^+^ T cells (Zhou et al., 2016), in contrast to the 2–3% of CD4^+^ and CD8^+^ T cells that co-expressed GrB and Perf consistent with an influenza-specific response (Kumar et al., 2017). In the absence of Perf to target the GrB response to killing virus-infected cells, GrB is released into the extracellular space causing collateral damage that is more widespread (Hirota et al., 2013). It has been established that extracellular GrB in the extracellular milieu regulates pro-inflammatory cytokine responses (Wensink et al., 2015), cleaves extracellular matrix proteins, and breaks down cell-cell junction proteins. Thus, in the extracellular milieu, GrB plays an important role in the loss of tissue function and integrity in the response to infection (Choy et al., 2004; Tschopp et al., 2006; Granville, 2010; Hendel et al., 2010; Russo et al., 2018; Turner et al., 2019; Matsubara et al., 2020), with high levels of GrB in bronchoalveolar lavage fluid that correlate with disease severity (Rasmuson et al., 2016). Our unpublished data have shown that the mean fluorescence intensity of GrB is 3-fold higher in GrB^+^Perf ^−^ CD8^+^ T cells than in GrB^+^Perf ^+^ CD8^+^ T cells, suggesting that these GrB^+^Perf ^−^ CD8^+^ T cells could be significant contributors to the inflammatory response and immunopathology of influenza virus infections in older adults.

The above findings become important when considering how the *ex vivo* GrB response to influenza vaccination changes the balance between CD8^+^ T cells that co-express GrB and Perf and those that do not express Perf when activated *ex vivo.* In this study, we showed that the *ex vivo* GrB response to influenza vaccination did not predict risk for LCII but was robustly stimulated following influenza infection, consistent with re-stimulation of T-cell memory. We have previously shown that this *ex vivo* GrB response to influenza A/H3N2 infection can effectively be re-stimulated with a subsequent influenza vaccination (McElhaney et al., 2009). Given that the frequencies of M1-and NP-specific T cells have been shown to represent correlates of protection, their inclusion in the influenza vaccine formulation administered in this study would appear to be an important contributor to the observed GrB response to vaccination. Even though the split-virus influenza vaccines administered in this clinical trial contain significant amounts of M1 and NP (Co et al., 2009), re-stimulation of CD8^+^ T cell memory relies on antigen cross-presentation by dendritic cells, a function that declines with age (Chougnet et al., 2015) and contributes to an already weak stimulus of memory CD8^+^ T cells. Our results are consistent with these findings in that, in general, there is a poor GrB response to vaccination against influenza A/H3N2 and B and thus influenza vaccination is unlikely to affect the balance between GrB^+^Perf^+^ CD8^+^ T cells and GrB^+^Perf^−^ CD8^+^ T cells responding to influenza infection and risk for influenza illness.

The strength of this study is the prospective evaluation of risk for LCII in a well-characterized cohort of older adults including documentation of LCII at the onset of illness. Frailty has been well characterized using a frailty index. We were able to demonstrate the difference in the *ex vivo* GrB response to vaccination compared to the specific response to natural A/H3N2 or B infection. Our study’s limitations are two-fold. First, we observed relatively few cases of LCII (∼4.5% incidence), which limited our detection power, and may raise uncertainty regarding our findings. A second limitation is that we are unable to identify the source of GrB activity produced in *ex vivo* virus-infected PBMCs, particularly the GrB released by GrB^+^Perf^−^CD8^+^ T cells, which, however, constitute the vast majority of GrB^+^ CD8^+^ T cells in older adults. When adjusted for levels of bGrB activity, we can demonstrate an iGrB response that is similar to our previous studies on correlates of protection and is consistent with the age-related decline in the CD8 T cell response to vaccination, but lacks the sensitivity needed as a robust biomarker offering a correlate of protection. Thus, our future focus will be on how these results translate to studies of the frequency of different T cell subsets responding to *ex vivo* influenza challenge, especially GrB-perforin double-positive CD8^+^ T cells.

## Data Availability

The raw data supporting the conclusions of this article will be made available by the authors, without undue reservation.
