# Supplementary material for: Granzyme B: A Double-Edged Sword in the Response to Influenza Infection in Vaccinated Older Adults
Source: Front Aging. 2021 Nov 11;2:753767. doi: 10.3389/fragi.2021.753767 (PMC9015675; doi:10.3389/fragi.2021.753767)
Supplement: Supplementary file 1 [file DataSheet1.docx]

**Supplemental Table 1.** Geometric mean hemagglutination inhibition (HAI) titres at each time point for LCII and non-LCII cases.

| **GeoMean (95% CI)** |  |  | **LCII at 10-20 weeks** | |
| --- | --- | --- | --- | --- |
|  | | **Non-LCII** | **A/H3N2** | **B** |
|  |  | **(N=582)** | **(N=17)** | **(N=9)** |
| **A/H3N2 HAI** | **Baseline** | 47.9 (44, 52.4) [4] | 41.7 (28.9, 60.1) [0] | 48.5 (26.2, 86.4) [0] |
|  | **4** | 156 (141, 173) [6] | 75.3 (51.1, 120) [0] | 154 (63.5, 388) [0] |
|  | **10** | 116 (106, 128) [19] | 60.1 (40, 90.4) [0] | 109 (40, 296) [0] |
|  | **20** | 96.2 (87.3, 106) [22] | 231 (139, 369) [0] | 109 (46.7, 296) [0] |
| **B HAI** | **Baseline** | 37.8 (35.3, 40.3) [4] | 46.1 (32, 70.8) [0] | 33 (23.3, 46.7) [0] |
|  | **4** | 78 (72.1, 83.6) [6] | 72.2 (51.1, 116) [0] | 61.1 (44.9, 86.4) [0] |
|  | **10** | 59.7 (55.5, 63.9) [19] | 56.6 (38.4, 86.8) [0] | 44.9 (30.5, 63.5) [0] |
|  | **20** | 54.3 (50.4, 58.1) [22] | 50.1 (34.7, 78.4) [0] | 89.8 (56.5, 148) [0] |

The count of missing data is shown in square brackets, and the time shown (ie. 4, 10, 20) represents weeks since vaccination.

**Supplemental Table 2.** Geometric Mean Granzyme B activity.

| **GeoMean (95% CI)** |  | **LCII at 10-20 weeks** | | |
| --- | --- | --- | --- | --- |
|  | **Total** | **A/H3N2** | **B** | **Null** |
|  | **(N=609)** | **(N=18)** | **(N=9)** | **(N=582)** |
| **GrB (H3N2) - Baseline** | 800 (762, 842) | 956 (762, 1202) | 1050 (770, 1406) | 792 (754, 834) |
| Missing | 5.00 (0.8%) | 0 (0%) | 0 (0%) | 5.00 (0.9%) |
| **GrB (H3N2) - 4-week** | 937 (890, 987) | 1007 (779, 1309) | 1230 (940, 1574) | 931 (885, 982) |
| Missing | 9.00 (1.5%) | 1.00 (5.6%) | 0 (0%) | 8.00 (1.4%) |
| **GrB (H3N2) - 10-week** | 901 (852, 951) | 1111 (811, 1529) | 1187 (942, 1582) | 892 (840, 942) |
| Missing | 25.0 (4.1%) | 1.00 (5.6%) | 0 (0%) | 24.0 (4.1%) |
| **GrB (H3N2) - 20-week** | 918 (873, 962) | 1447 (1198, 1745) | 1327 (976, 1724) | 899 (854, 948) |
| Missing | 30.0 (4.9%) | 1.00 (5.6%) | 0 (0%) | 29.0 (5.0%) |
| **GrB (B) - Baseline** | 659 (625, 695) | 767 (615, 967) | 803 (595, 1038) | 654 (617, 691) |
| Missing | 16.0 (2.6%) | 1.00 (5.6%) | 0 (0%) | 15.0 (2.6%) |
| **GrB (B) - 4-week** | 811 (773, 856) | 826 (650, 1051) | 975 (722, 1357) | 809 (766, 853) |
| Missing | 17.0 (2.8%) | 1.00 (5.6%) | 0 (0%) | 16.0 (2.7%) |
| **GrB (B) - 10-week** | 745 (702, 788) | 755 (569, 984) | 980 (768, 1258) | 742 (698, 787) |
| Missing | 30.0 (4.9%) | 1.00 (5.6%) | 0 (0%) | 29.0 (5.0%) |
| **GrB (B) - 20-week** | 783 (740, 824) | 767 (651, 904) | 1683 (1272, 2194) | 774 (734, 815) |
| Missing | 40.0 (6.6%) | 1.00 (5.6%) | 0 (0%) | 39.0 (6.7%) |
| **bGrB** | 129 (117, 141) | 201 (120, 336) | 147 (72.7, 305) | 127 (114, 141) |
